# Sex differences underlying orofacial varicella zoster associated pain in rats

**DOI:** 10.1186/s12883-017-0882-6

**Published:** 2017-05-17

**Authors:** Crystal Stinson, Mohong Deng, Michael B Yee, Larry L. Bellinger, Paul R. Kinchington, Phillip R. Kramer

**Affiliations:** 1 Department of Biomedical Sciences, Texas A&M University College of Dentistry, 3302 Gaston Avenue, Dallas, TX 75246 USA; 2 Dept Ophthalmology and of Molecular Microbiology and Genetics, 203 Lothrop St., Pittsburgh, PA 15213 USA; 3 Department of Oral and Maxillofacial Surgery, The State Key Laboratory Breeding Base of Basic Science of Stomatology & Key Laboratory of Oral Biomedicine Ministry of Education, School & Hospital of Stomatology, Wuhan University, Wuhan, People’s Republic of China

**Keywords:** Post-herpetic neuralgia, Shingles, Chickenpox, Trigeminal ganglia, Orofacial

## Abstract

**Background:**

Most people are initially infected with varicella zoster virus (VZV) at a young age and this infection results in chickenpox. VZV then becomes latent and reactivates later in life resulting in herpes zoster (HZ) or “shingles”. Often VZV infects neurons of the trigeminal ganglia to cause ocular problems, orofacial disease and occasionally a chronic pain condition termed post-herpetic neuralgia (PHN). To date, no model has been developed to study orofacial pain related to varicella zoster. Importantly, the incidence of zoster associated pain and PHN is known to be higher in women, although reasons for this sex difference remain unclear. Prior to this work, no animal model was available to study these sex-differences. Our goal was to develop an orofacial animal model for zoster associated pain which could be utilized to study the mechanisms contributing to this sex difference.

**Methods:**

To develop this model VZV was injected into the whisker pad of rats resulting in IE62 protein expression in the trigeminal ganglia; IE62 is an immediate early gene in the VZV replication program.

**Results:**

Similar to PHN patients, rats showed retraction of neurites after VZV infection. Treatment of rats with gabapentin, an agent often used to combat PHN, ameliorated the pain response after whisker pad injection. Aversive behavior was significantly greater for up to 7 weeks in VZV injected rats over control inoculated rats. Sex differences were also seen such that ovariectomized and intact female rats given the lower dose of VZV showed a longer affective response than male rats. The phase of the estrous cycle also affected the aversive response suggesting a role for sex steroids in modulating VZV pain.

**Conclusions:**

These results suggest that this rat model can be utilized to study the mechanisms of 1) orofacial zoster associated pain and 2) the sex differences underlying zoster associated pain.

## Background

Varicella zoster virus (VZV) is a human herpesvirus that infects a majority of the unvaccinated population worldwide. While recovery from varicella “chickenpox” occurs with lifelong immunity the virus persists in a latent state in the nuclei of neurons within sensory and autonomic ganglia across the entire neuro-axis. VZV chromatin remains in the infected cells (extrachromosomal) but viral protein expression and virus production remain repressed [1–4]. However, reactivation from latency occurs in about one third of VZV positive individuals to cause herpes zoster (HZ), with the incidence of HZ increasing with age and declining immune status. The skin rash of HZ is often large but geographically restricted to a dermatome infiltrated by one or two specific ganglia. Reactivation is accompanied by extensive intra-ganglionic spread, infection of multiple neurons and numerous inflammatory and physiological processes that contribute to the development of pain. Almost all HZ is associated with acute pain, with up to 90% of HZ patients seeking prescription level pain relief [5]. However, some 30% of HZ patients report a chronic pain state termed post herpetic neuralgia (PHN), defined as pain lasting more than 3 months after the resolution of the skin lesions [6–8]. A fraction of PHN patients may suffer pain more than one year after other disease symptoms dissipate [9]. PHN is debilitating, difficult to treat, and can have secondary long term consequences that can greatly affect quality of life. In the absence of the current HZ vaccine approximately 1 million people annually in the US and about 5% of the total population will suffer from PHN [9–11]. The majority of HZ sufferers are age 60 years or older [12, 13]. Given that the number of people 60 years of age or older is one of the fastest growing and is expected to double in the next 50 years [9], HZ and the associated pain will be an ever increasing problem in the future. While the HZ vaccine reduces the incidence of HZ by 51% and PHN by 68%, the vaccine remains to be widely used largely due to cost/reimbursement issues, reluctance to vaccination, and ignorance of both the target population and their physicians. [11]. Even if every candidate receives the vaccine (which is far from being achieved) the US would still have some half million HZ cases each year with many suffering from PHN. As such, better prevention and therapy for both zoster and PHN are an unmet need.

It has been difficult to model HZ and PHN because no animal model shows reactivation of latent VZV. In general, VZV is considered human specific, for reasons not yet clear. However it has been shown that VZV injected into the footpad of either Wistar or Sprague Dawley rats results in measurable hypersensitivity that is similar to that in humans with PHN [14–20]. The footpad animal model has been developed as a platform for one, mechanistic studies of chronic pain after VZV infection of dorsal root ganglia and two, for drug evaluation focused on HZ associated pain relief [20, 21]. Treatment of these animals with antivirals, opioids or NSAIDS mirrors HZ patients treated with these same drugs, in that, zoster associated pain does not respond to antivirals, but does respond to treatment with gabapentin or sodium channel blockers [16, 17, 20, 21].

Given that the rat footpad model has been used to study the mechanism by which VZV associated pain develops and can be used for pre-clinical screening of analgesic drugs [17, 21] we considered whether the model can be extended to orofacial pain that follows VZV reactivation in the trigeminal ganglia. Extension of the model to the orofacial region would be significant because the trigeminal ganglia of human cadavers have the highest latent VZV load, and up to 1 in 5 HZ cases occurs in the orofacial or naso-ocular region [22, 23]. Importantly, females report HZ associated pain 3.75 times more often than men, and HZ associated pain has been reported to be 36% higher in females [24, 25]. The basis for this disparity is not understood. Currently, neither the orofacial effects nor the sex differences have been determined in any zoster associated pain model. In this report, an animal model for zoster associated orofacial pain was characterized that could be utilized first, to study the mechanisms of HZ associated pain in the orofacial region and second, to study the mechanisms resulting in women reporting HZ pain more often than men.

## Methods

### Cells and viruses

VZV Parent of Oka (pOka), a varicella isolate that was the parent strain used to derive the current VZV vaccine strain, was used in all this work. pOka VZV was previously shown to induce chronic pain indices in the rat model [15]. Virus was used at less than 12 passes beyond receipt from M. Takahashi (Osaka University, Japan) and propagated as detailed previously [26, 27] on the VZV permissive MeWo human cell line (ATCC, Manassas, VA). Cells and virus were grown in Minimal Essential Media (MEM) supplemented with 10% fetal bovine serum (FBS) and antibiotic and antimycotic mixture (Sigma, St. Louis, MO). VZV was prepared to high titer as previously detailed [15] on confluent monolayers of MeWo cells infected at ~0.1 plaque forming units (pfu)/cell. Virus infected cells incubated at 35 °C for 48–72 h post infection were harvested by trypsin digestion, and optimal VZV cell viability was maintained by slow freezing aliquots in MEM containing 20% FBS and 10% dimethyl sulfoxide (Sigma, St. Louis, MO), followed by storage under liquid nitrogen. Virus titers of frozen stocks were assessed by an infectious center assay. Uninfected cell equivalents were prepared using similar treatments. All virus and cellular stocks were provided by Dr. Kinchington’s laboratory.

### Animal studies

All work with animals was approved by the Texas A&M University College of Dentistry Institutional Animal Care and Use Committee. Male and female Sprague-Dawley or Long Evans rats (280–300 g) were purchased from Envigo (Indianapolis, IN) and kept on a 14:10 light/dark cycle. The rats were given food and water ad libitum. After a 4 day acclimation period, experiments were carried out in accordance with NIH regulations on animal use. Vaginal smears were performed daily on female rats to determine the stage of the estrous cycle. The body weights were recorded weekly. Six experiments using different batches of rats (see Table 1) were completed to measure behavior after injection of a high (1900 pfu/μl) or low (650 or 360 pfu/μl) dose of VZV (pOka strain). Male Sprague-Dawley rats were used in the experiments unless otherwise indicated.

**Table 1:** Experiments completed for each study (i.e. set of animals)

Study	Experiment	Groups	Number of animals (male Sprague Dawley rats unless otherwise indicated)
Study #1	Thermal and mechanical hypersensitivity assay (Figure 3). Neurite retraction study using PGP 9.5 (*n* = 3/group randomly selected, Figure 2).	Mewo (control) Poka (VZV)	*n* = 10 *n* = 10
Study #2	Place escape/avoidance paradigm (Figure 4). VZV infection detected with IE62 marker (Figure 1).	PBS (vehicle) Mewo (control) Poka (VZV virus)	*n* = 6 *n* = 6 *n* = 6
Study #3	Gabapentin assay (Figure 5).	Mewo/vehicle Mewo/gabapentin Poka/vehicle Poka/gabapentin	*n* = 5 *n* = 5 *n* = 5 *n* = 5
Study #4	Sex differences study comparing males and females (Figure 6).	Mewo/female Mewo/male Poka/female Poka/male	*n* = 24 *n* = 7–15 *n* = 25 *n* = 7–15
Study #5	OVX females as compared to males (Sprague Dawley and Long Evans, Figure 7). VZV infection detected with IE62 marker (Sprague-Dawley rats, no figure).	Mewo/male Mewo/female Mewo/OVX female Poka/male Poka/female Poka/OVX female	*n* = 5 *n* = 4 *n* = 4 *n* = 7 *n* = 6 *n* = 6 *n* = 5 (Long Evans) for all treatment groups
Study #6	Female estrous cycle study (Figure 8).	Mewo diestrus Mewo proestrus/estrus Poka diestrus Poka proestrus/estrus	*n* ≥ 8 *n* ≥ 8 *n* ≥ 8 *n* ≥ 8

### Training for thermal and mechanical hypersensitivity assay

In the first experiment the animals were trained to obtain reward using the orofacial test chamber from Ugo Basile (Italy) [28–30]. The reward was a sweetened condensed milk mixture, 100 ml milk and 200 ml water. Drinking time was measured by using an infrared beam that detects when the animal has placed their nose into the spigot area to access the drinking supply. No thermal testing or filament testing was performed during the initial one week training period where the animals were placed in the chambers daily for 10 min to obtain a reward. No filaments or thermal coils were present in the training period. Next, the pre-testing training period continued another two weeks and included a daily 12 h fast followed by 10 min of familiarization with the chamber environment with the reward inaccessible and 10 min of active testing with the reward accessible. Training was successful when animals consistently drank milk for 300 ± 50 s out of a 600 s testing period with thermal coils or filaments in place at the spigot opening. All testing was done in the beginning of the dark phase with red lights.

To measure thermal and mechanical hypersensitivity induced by VZV the animals were anesthetized briefly with 2% isoflurane using a 2 l per minute flow of air, the whisker pad of Sprague Dawley rats were then inoculated with uninfected MeWo cells, or the same number of MeWo cells infected with (1900 pfu/μl) VZV by injecting a 100 μl volume subcutaneously. The animals were mobile within 5 min of the injection. The whisker pad was chosen as the injection site because it has a large number of sensory terminals that would allow for infection of VZV. Testing was completed either before the injection (pre-injection) or one week after injection. Testing continued once every seven days thereafter. For the thermal test the coils of the test chamber were set to 45 degrees Celsius. For the filament test, twelve nickel/titanium/chromium wires 0.16 mm (0.006″) in diameter were placed evenly around a circular opening 3.5 cm in diameter. Each wire projected 1.5 cm into the center of the opening. The weekly test schedule assessed baseline responses without either a thermal coil or a filament on the first day of each week, followed by thermal testing the following day and filament testing on the next day. Testing was done for a 6 week period.

### Place escape/avoidance paradigm (PEAP) testing in male rats

In a second experiment behavioral responses were measured using the PEAP test. Testing was completed by a tester blinded to the treatment. For this experiment the whisker pad of a second group of male rats was injected with a high dose of VZV (1900 pfu/μl), or equivalent control MeWo cells or PBS (100 μl volume). Rats were anesthetized as described previously for the injection. At 7 days post inoculation the animals were placed in a 30 cm X 30 cm X 30 cm acrylic box in which half the box was covered in black cloth. This test chamber was modeled from the PEAP test performed and detailed by the Fuchs lab [31]. PEAP testing has been used in inflammatory and neuropathic pain models, used in both sexes and various strains and ages of rats as a means of measuring affective aspects of pain [31–37]. Each individual rat was placed into the chamber; rodents being nocturnal prefer the dark side of the chamber. The study was then initiated by stimulating the face with a 60 g filament every 15 s; rats on the dark side of the chamber receive a stimulus at the injected side, whereas rats with their head on the clear side of the chamber were stimulated on the contralateral non-injected side. The region receiving the stimulus was in the region below the eye and caudal to the whisker pad, which is innervated by the second branch of the trigeminal ganglia. Because sensory neurons of the second branch also innervate the whisker pad, we expected sensitivity of the face around the eye [38]. The time spent on the dark side of the box was recorded in 5 min bins and testing was performed for a total of 30 min on the test day. Testing was performed one day a week and continued for 8 weeks.

### VZV injection in male rats to investigate analgesia efficacy

A third group of male rats were injected at the whisker pad with either a low dose of VZV (360 pfu/μl) or control MeWo cells. One week after VZV injection the rats were given a 3.5 ml gavage of either 0.9% saline, or 50 mg/ml gabapentin (Amneal Pharmaceuticals, Branchburg, NJ). PEAP testing was performed 30 min after gavage. The effect of analgesics can be undetectable “swamped out” if the nociceptive response is too great thus, the lowest dose of VZV known to elicit a behavioral response was implemented before administration of gabapentin.

### Sex differences in VZV induced aversive behavior

In a fourth set of studies intact male and intact female rats were injected with 650 pfu/μl of VZV or equivalent control MeWo cells. PEAP testing was performed once a week over a six week period.

### Compare VZV induced aversive behavior of ovariectomized females to intact males

Female rats were ovariectomized to simulate menopause because a majority of women with HZ pain will be in a post-menopausal state and post-menopausal women report HZ associated pain more than older men. In this fifth study intact male, intact female and OVX females from two strains of rat were utilized (Sprague Dawley and Long Evans rats). Sprague Dawley rats were inoculated with 650 pfu/μl of VZV and Long Evans rats were inoculated with 360 pfu/μl of VZV. PEAP testing was performed 7 days after injection and continued once a week for 2 weeks. Long Evans rats were reported by Envigo to be less docile than Sprague Dawley rats and thus, the Long Evans rats were injected with a lower dose of VZV as compared to the Sprague Dawley rats in Study #5. This lower dose was expected to compensate for the greater inherent activity of Long Evans rats during the behavioral testing.

### Ovariectomy surgery

Animals were anesthetized with 2% isoflurane using a 2 l per minute flow of air. The anesthetized female was placed in ventral recumbency with their tail toward the surgeon. The dorsal surgical area was shaved and swabbed with surgical scrub. A short dorsal midline skin incision was made halfway between the caudal edge of the ribcage and the base of the tail. An abdominal muscle wall incision was made lateral to the spine to provide access to the peritoneal cavity. The ovary and the oviduct were exteriorized through the muscle wall incision. A sterile silk ligature was placed around the oviduct. Each ovary and part of the oviduct was removed with a single cut through the oviduct near the ovary. The remaining tissue was replaced into the peritoneal cavity and the opening sutured. The ovary on the contralateral side was removed in a similar manner and the animal was given a 2 mg/kg dose of nalbuphine.

### Compare VZV induced aversive behavioral response over estrous cycle

In a sixth set of studies vaginal smears were taken immediately after PEAP testing each week using the intact females from the fourth study. The groups were then divided based on the phase of the estrous cycle.

### Immuno-fluorescent staining

After testing for hypersensitivity or aversive behavior the rats were sacrificed and perfused for immunohistochemistry. Whisker pad tissue was collected from the first study and trigeminal ganglia were collected from the second study and fifth study (see Table 1). Because VZV infection, as determined by the presence of IE62, showed greater levels of IE62 at 1 month and 18 months post infection versus one week post infection [39]. Thus, we expected greater VZV infection and neurite retraction in the first and second studies that were of longer duration. Tissue in study five was analyzed to determine if the sex differences were due to differences in VZV infection.

After administering 100 mg/kg ketamine/10 mg/kg xylazine the rats were perfused with 9% sucrose followed by 4% paraformaldehyde. Fixed tissues were stored in 25% sucrose, frozen, cryo-sectioned and the 20 μm sections placed on Histobond slides (VWR international, Radnor, PA). The tissue was then blocked with a PBS solution containing 5% normal goat serum and 0.3% Triton-X 100 for 2 h at room temperature. The slides were incubated in a primary antibody solution overnight at 4 °C. Primary antibodies consisted of a 1:200 dilution of the mouse monoclonal IE62 antibody (Acris Antibodies Inc., San Diego, CA) and a 1:250 dilution of the rabbit monoclonal NeuN antibody (Abcam, San Francisco, CA); or a 1:500 dilution of the rabbit polyclonal PGP9.5 antibody (BioRad, Raleigh, NC). Primary antibodies were diluted with PBS, 5% BSA and 0.3% Triton X-100. After incubation in primary antibody the slides were rinsed three times in PBS and Triton-X 100 for a total of 45 min and placed for 2 h in a 1:500 dilution of secondary antibody in PBS and 0.3% Triton X-100. Secondary antibodies included a mixture of goat anti-mouse 568 and goat anti-rabbit 488 1:500 dilution (Invitrogen, Carlsbad, CA). For the PGP9.5 antibody staining a 1:500 dilution of a goat anti-rabbit 568 secondary was used. After rinsing the slides three times in PBS for a total of 45 min, the slides were then mounted with Fluoromount-G mounting medium containing Hoechst 33,342 stain (Electron Microscopy Sciences, Hatfield, PA). The fluorescent signal was imaged using a Nikon fluorescent microscope and NIS-Elements imaging software and a Photometrics CoolSnap K4 CCD camera (Roper Scientific, Inc., Duluth, GA).

### Cell counting

Quantitation of IE62 staining was completed by a blinded reviewer counting the number of IE62 and NeuN positive cells within a randomly chosen 0.5 mm circular field near either the V3 branch or the V1/V2 branches. The neurons were primarily circular in shape and were divided into small C-fibers (<23 μm diameter), A-δ medium-sized (23–32 μm diameter) and A-α/β large neurons (32 > μm diameter) based on findings from the dorsal root ganglia [40]. Or the counts for all NeuN positive cells were combined. Three randomly chosen trigeminal ganglia sections were analyzed for each animal. Three animals were randomly chosen from each treatment group.

Measurement of neurite infiltration was performed as recently detailed [15]. Sections were counted for PGP9.5 positive neurites in coronal whisker pad sections, and were determined by a reviewer blinded to the treatment group. All PGP9.5 positive filaments that crossed the stratum basale/dermis border terminating between the surface of the skin and this border were counted; counts were determined along each mm of skin in the section. Neurite counts were completed by choosing a single random field ~5 mm in length along the stratum basale/dermis border on 10 randomly chosen sections taken from three randomly chosen animals from each treatment group. The number of filaments counted per mm of skin for each section was reported.

### Statistical analysis

Data was analyzed with ANOVA and each time point was considered a replication. Dependent variables included the hypersensitivity measurements, PEAP data or cell counts and the independent variables were treatment, sex, cell size and trigeminal ganglia branch. When a significant effect was observed Bonferroni post-hoc tests were completed (Prism 5.04, GraphPad Software, La Jolla, CA, or Abstat, Anderson Bell Corp, Arvada CO). When two groups were being compared a two-tailed Mann-Whitney t-test was performed. The number of animals per group is outlined in Table 1.

## Results

### VZV accesses the trigeminal ganglia after whisker pad inoculation

**Fig. 1:**
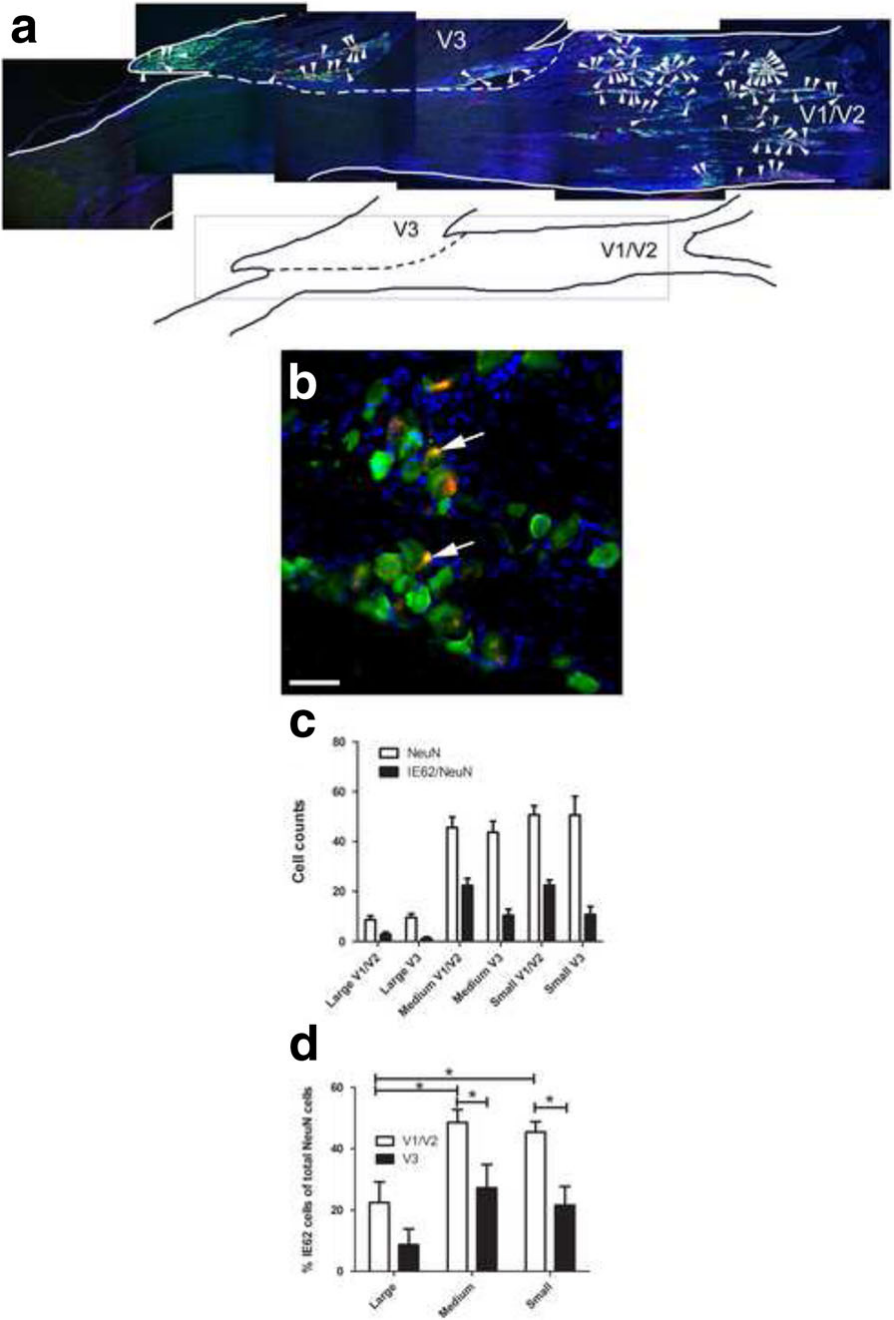
The VZV marker IE62 was expressed in neurons of the trigeminal ganglia after injecting the whisker pad with virus. Sprague Dawley rats from the second study were injected with 100 μl of 1900 pfu/ μl VZV and tissues were collected 8 weeks following injection. Immunofluorescent detection of IE62 (*red*) and the neuronal marker NeuN (*green*) was completed on fixed frozen sections. *Blue* was nuclear Hoeschst stain. A sagittal section of the trigeminal ganglia is shown in panel **a** (low magnification) and in **b** (high magnification). In panel **a** the boxed area within the cartoon shows the stained region of the trigeminal ganglia. *Yellow cells* co-labeled with IE62 and NeuN in panel **a** are indicated by arrowheads. The branches of the trigeminal ganglia are labeled V1, V2 and V3. The V3 border is represented by a *dotted line*. In panel **b**, co-localization of NeuN and IE62 was shown in *yellow* and cells were indicated by *arrows*. Bar equals 50 μm. Panel **c** shows the total number of NeuN and double stained NeuN and IE62 positive cells in the trigeminal ganglia. Panel **d**, the percent of NeuN/IE62 double labeled cells in the total NeuN positive population was calculated for the neurons in different regions of the trigeminal ganglia. Small neurons were <23 μm in diameter, medium-sized neurons were 23–32 μm in diameter and large neurons were >32 μm in diameter. An asterisk indicates *p* < 0.05, *n* = 6. Values shown are the mean and SEM

In the virus injected group NeuN positive cells co-localize with IE62 within the trigeminal ganglia (Fig. 1a and b). IE62 is one of the first proteins translated from the VZV genome after infection. IE62 is an essential intermediate early gene that regulates expression of most VZV genes and the production of VZV localizes to both the nucleus and cytoplasm [41]. From the image the majority of IE62 was in the neuronal cell population but an analysis of other cell types was not completed. In these male rats IE62 staining was in neurons of all sizes (Fig. 1c) but there was a significantly greater percent of IE62 in the smaller neurons, F(2, 68) = 8.02, *p* < 0.001 and this staining was significantly greater in the region innervated by the V1/V2 branch, F(1, 68) = 17.09, *p* < 0.001 (Fig. 1d). Note that the whisker pad injection site is innervated by the V2 branch of the trigeminal ganglia. No IE62 stain was observed in the MeWo or PBS groups (data not shown).

### As in humans virus infection induces neurite retraction

**Fig. 2:**
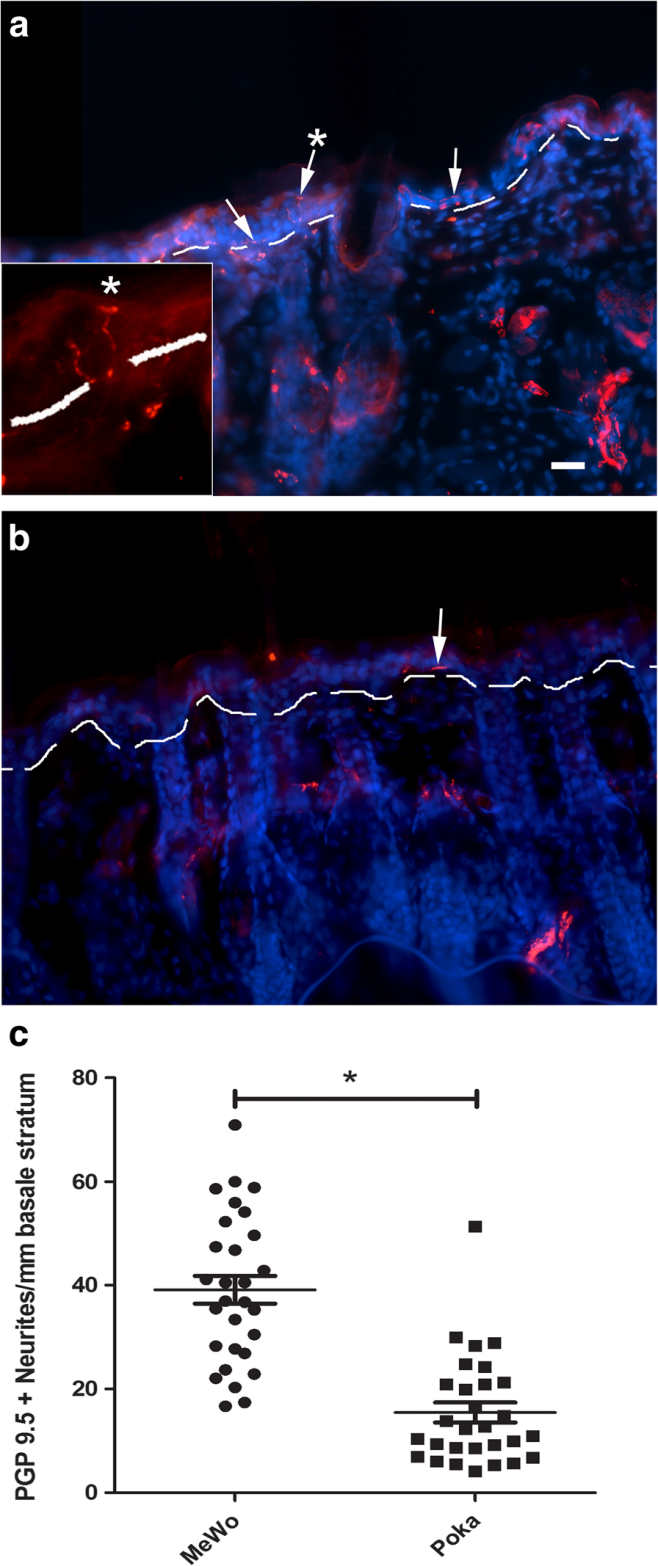
VZV induced peripheral retraction of PGP9.5 positive neurites in the skin of the whisker pad. At six weeks after whisker pad injection with either MeWo cells (**a**) or MeWo cells containing VZV (**b**) male Sprague Dawley rats from the first study were sacrificed and the whisker pad skin processed for analyses. Ten 20 μm coronal sections were cut from each injected whisker pad. Representative images for MeWo injected (**a**) and pOka injected (**b**) rats are shown after staining with the PGP9.5 antibody (*red*), cell nuclei are stained *blue*. The border between the stratum basale and dermis is shown as a white dotted *line*. *Arrows* point to PGP9.5 positive filaments. Asterisk above the *arrow* indicates enlarged region within box. Bar = 50 μm. In panel **c**, each data point on the histogram shows the average number of PGP9.5 positive neurites per mm that terminate above or cross the basale/dermis border for each section. There were ten sections per rat and three rats per treatment group. An asterisk indicates *p* < 0.05. Mean and SEM are shown

Skin biopsies of zoster patients suffering PHN have reduced peripheral innervation of the skin in comparison to zoster patients not suffering from PHN [42, 43]. This was also recently found in the rat footpad PHN model [44]. We therefore examined peripheral innervation of neurites at the whisker pad, using PGP9.5 staining to identify and visualize neurites (Fig. 2a, b). Rats showing hypersensitivity due to VZV injection (pOka strain) had a significantly reduced peripheral innervation of the whisker pad skin (Fig. 2c).

### Development of pain behaviors after VZV inoculation of the whisker pad

**Fig. 3:**
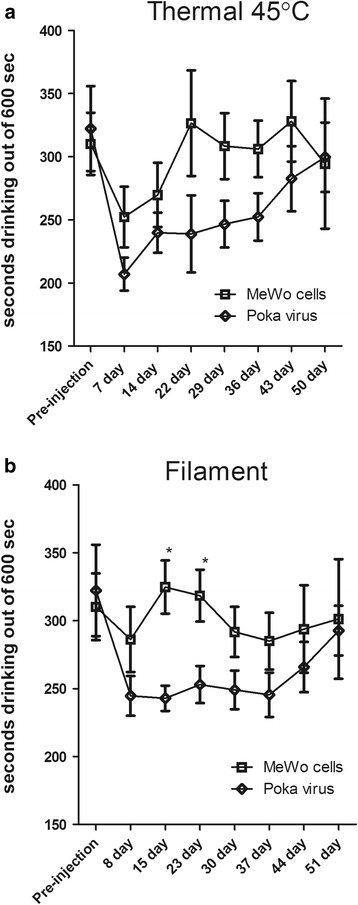
Thermal and mechanical hypersensitivity increased after inoculating the whisker pad with VZV. Male Sprague Dawley rats were injected with either MeWo cells or MeWo cells infected with 1900 pfu/μl VZV. Testing was performed for 50–51 days post injection. Testing was completed using the orofacial test module from Ugo Basile. Thermal testing (panel **a**) was completed at 45 °C and filament testing (panel **b**) was performed with a custom circular opening containing 12 filaments. Fewer seconds drinking is an indicator of greater hyperalgesia or allodynia. Asterisk indicates a significant difference (*p* < 0.05) between the MeWo and VZV (pOka) treatment groups. There were 10 animals per treatment group and values are the mean and SEM

Whisker pad injection of MeWo cells infected with VZV (pOka strain) revealed the development of some facial thermal hypersensitivity F(1170) = 7.1, *p* < 0.01 as compared to control uninfected cells (Fig. 3a). There was no significant interaction of treatment and time F(1170) = 0.63, *p* = 7.2. Testing for mechanical hypersensitivity showed a significant response over the 51 day testing period F(1170) = 16.3, *p* < 0.0001 with no significant interaction between treatment and time F(1170) = 1.7, *p* = 0.1 (Fig. 3b). Post-hoc testing revealed a significant difference between the pOka and MeWo treatment groups at days 15 and 23 (Fig. 3b).

**Fig. 4:**
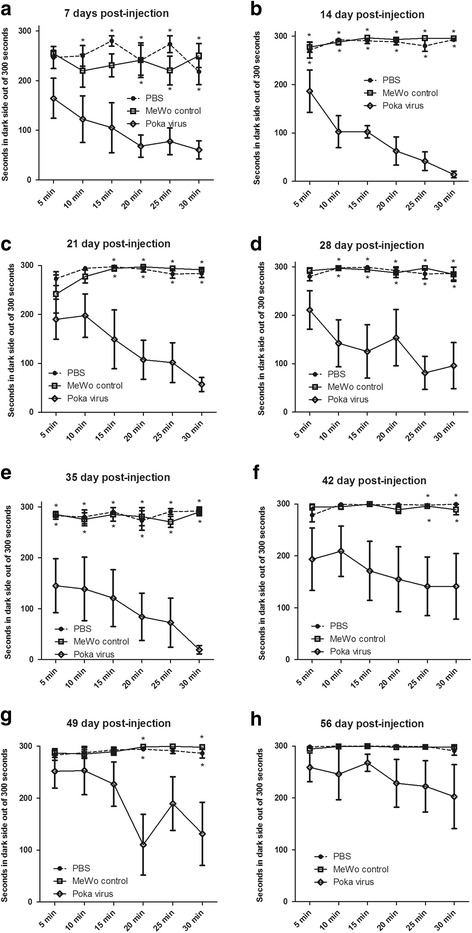
VZV increased aversive behavior as measured by the Place Escape/Avoidance Paradigm (PEAP) test. PEAP was completed on males whose whisker pad was injected with either PBS (vehicle), MeWo cells or MeWo cells infected with VZV (1900 pfu/ μl). The PEAP test was performed for eight weeks and each panel shows the data for a different week; panel **a** shows week 1, panel **b** week 2, panel **c** week 3, panel **d** week 4, panel **e** week 5 and panel **f** week 6, panel **g** week 7 and panel **h** week 8. The upper row of asterisks indicate a significant difference (*p* < 0.05) between the PBS and pOka treatment groups. Asterisks on the lower row indicate a significant difference (*p* < 0.05) between MeWo and pOka treatment groups. There were 6 animals per treatment group

In addition to measuring thermal and mechanical hypersensitivity a PEAP test was completed after whisker pad injection. In this assay, reduced time on the dark side during the stimulation phase indicates a greater affective pain response [31]. The response to VZV injection showed a remarkable difference that was significant for 7 weeks in comparison to control (Fig. 4). Significance was observed in week one, F(2,75) = 19.9, *p* < 0.001 (Fig. 4a); week two F(2,75) = 196.1, *p* < 0.001 (Fig. 4b); week three F(2,75) = 31.3, *p* < 0.001 (Fig 4c); week four F(2,75) = 14.7, *p* < 0.001 (Fig. 4d); week five F(2,75) = 20.1, *p* < 0.001 (Fig. 4e); week six F(2,75) = 5.2, *p* < 0.05 (Fig. 4f); and week seven F(2,75) = 6.4, *p* < 0.01 (Fig. 4g). The effect of virus in week eight was no longer significant, F(2,75) = 2.4, *p* = 0.12 (Fig, 4h). A significant interaction between the PBS, MeWo and virus pOka groups was observed in weeks two F(10,75) = 5.8, *p* < 0.001 (Fig. 4b) and three F(10,75) = 2.5, *p* < 0.05 (Fig. 4b). No significant interaction was observed in the other weeks (data not shown). Post hoc testing demonstrated a difference between the VZV and control groups for 7 out of the 8 weeks (Fig. 4a-g) but after eight weeks, there was no significant difference (Fig. 4h). These results strongly suggest the development of a virus specific pain response that did not develop after injecting uninfected cells or PBS. The PEAP assay was found to be more indicative and sensitive to changes resulting from virus injection and thus, the PEAP assay was continued throughout the rest of the following studies. During the first 5 min there was no significant difference in the time spent on the light side of the box when comparing groups in the first week to following weeks. This data suggests the animals do not recall the events of previous week when the 30 min testing periods are separated by a 7 day period of no testing.

### VZV induced orofacial pain responds to gabapentin.

**Fig. 5:**
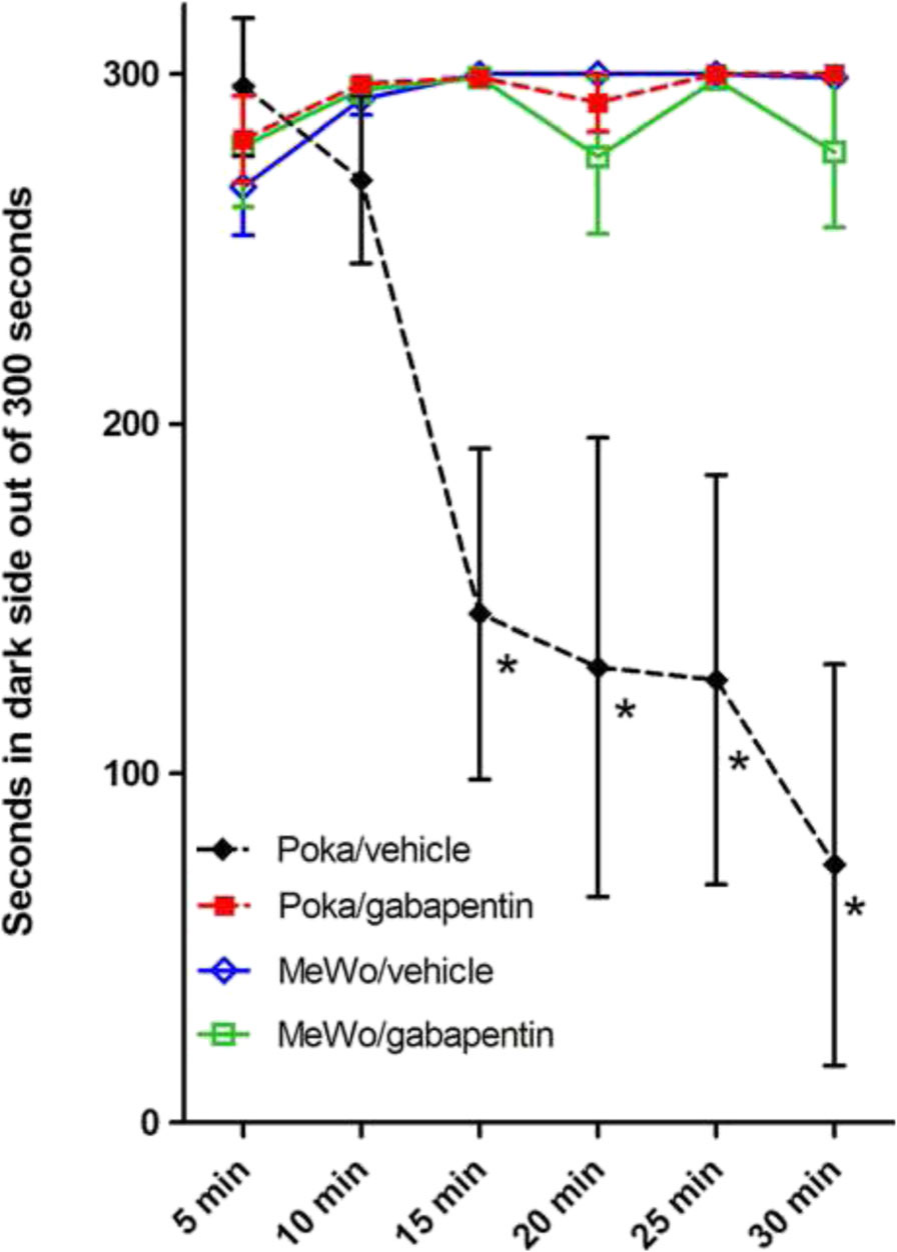
Gabapentin relieves the VZV induced aversive response. The PEAP test was assessed at 7 days post infection in rats whose whisker pad was injected with either MeWo cells or MeWo cells infected with a low dose of VZV (360 pfu/ μl). An asterisk indicates a significant difference between the VZV pOka/vehicle group and the VZV pOka/gabapentin group, as well as, a significant difference between the Mewo/vehicle group and the pOka/vehicle group. There were 5 animals per treatment group

Seven days after injecting the whisker pad (360 pfu/μl VZV), gabapentin was administered by oral gavage. Injection of virus induced a pain response F(2, 171), 13.41, *p* < 0.01 (Fig. 5), however gabapentin significantly F(2, 171) = 6.75, *p* < 0.01 reduced this response 30 min after administrating gabapentin. Post-hoc testing demonstrated that gabapentin significantly reduced the response at the 15, 20, 25 and 30 min testing periods (Fig. 5). A significant virus/drug interaction was observed F(2, 171) = 7.77, *p* < 0.01. No significant differences were observed between the MeWo/vehicle and MeWo/gabapentin treatment groups.

### Sex based differences in the development of VZV induced pain at the whisker pad.

**Fig. 6:**
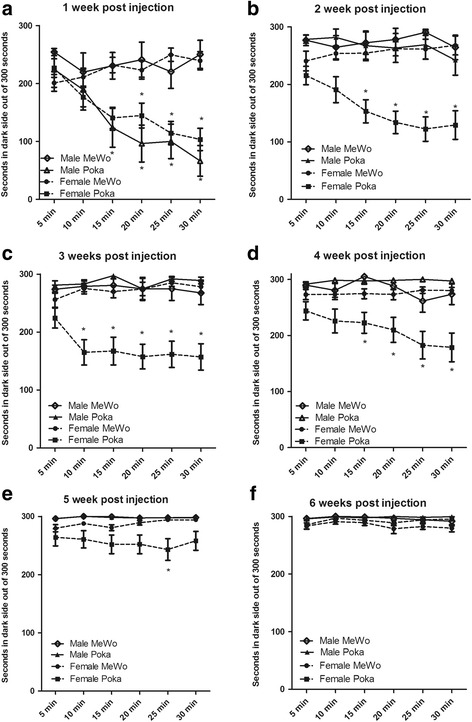
Sex differences in VZV induced aversive response. Male and female whisker pads were injected with either MeWo cells or MeWo cells infected with 650 pfu/ μl VZV. PEAP testing was completed weekly over six weeks, each panel shows data for a different week. In panel **a** the upper row of asterisks indicate a significant difference between the Female MeWo and Female pOka treatment groups and the lower row of asterisks indicate a significant difference between the Male MeWo and Male pOka treatment groups. In panels **b**-**f** an asterisk indicates a significant difference (*p* < 0.05) between the Female MeWo and Female pOka treatment groups. There were 24 control and 25 virus infected females and the number of males in both the MeWo and pOka treatment groups was 7 for week one, 10 for week two and 15 for weeks three, four, five and six

We carried out a comparison of the pain response in male and female rats to determine if sex differences exist in this virus model. One week after VZV injection, the PEAP response significantly increased in the virus versus MeWo control group F(1, 395) = 19.1, *p* < 0.001 (Fig. 6a) in both sexes of rats, and there was no significant difference between the sexes F(1, 395) = 0.03, *p* = 0.85. In contrast, two weeks F(1, 443) = 12.1, *p* < 0.001 and three weeks after injection F(1, 473) = 11.0, *p* < 0.01 there was a significantly greater pain response in the virus versus control group for the females but not the males (Fig. 6b and c). Moreover, the difference between male and female virus groups was significant in week two F(1443) = 16.6, *p* < 0.001 (Fig. 6b), three F(1, 473) = 15.4, *p* < 0.001 (Fig. 6c), four F(1, 473) = 10.6, *p* < 0.01 (Fig. 6d) and five F(1, 473) = 8.4, *p* < 0.01 (Fig. 6e). This sex difference was no longer significant in week 6 (Fig. 6f). No significant interaction between sex and virus was observed in week one and five but in week two, three and four a significant interaction was observed F(1443) = 4.9, *p* < 0.05, F(1, 473) = 16.8, *p* < 0.001, F(1, 473) = 11.0, *p* < 0.01, respectively.

Our data also show that a dose response to VZV occurred, in that a lower responses was detected after injecting a lower VZV dose of 650 pfu/ml (Fig 6) contrasted to the more significance responses seen with the higher VZV dose in Fig. 4 (1900 pfu/ μl). With the lower VZV dose, significant behavioral responses were much shorter in male rats, lasting for only one week (Fig. 6), while the pain response to a higher dose lasted for seven weeks (Fig. 4).

### Pain was reduced in male rats versus female rats in a post-menopausal state

**Fig. 7:**
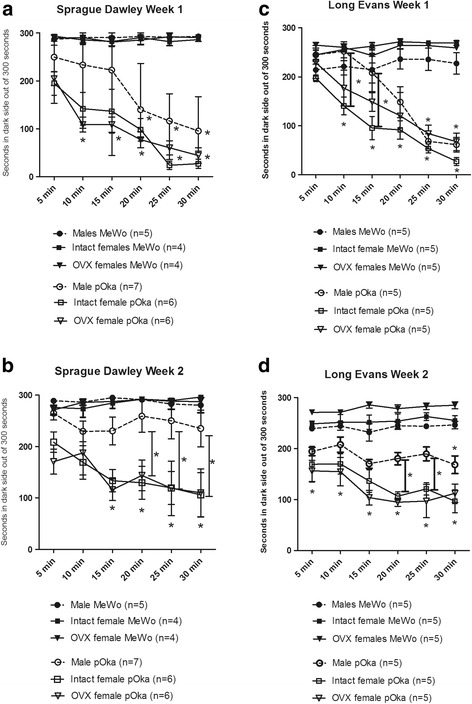
OVX female rats show an increased VZV induced aversive response as compared to males. The whisker pads of male and intact or ovariectomized (OVX) female rats were injected with either MeWo cells or MeWo cells infected with 650 pfu/μl VZV (Sprague Dawley rats, panels **a** and **b**) or 360 pfu/μl VZV (Long Evans rats, panels **c** and **d**). PEAP testing was completed weekly over two weeks. The lower row of asterisks indicate a significant difference between the Female MeWo and Female pOka treatment groups (both intact and OVX) and the upper row of asterisks indicate a significant difference between the Male MeWo and Male pOka treatment groups. In panels **b** and **d** the bar with an asterisk indicates a significant difference (*p* < 0.05) between the Male pOka and the intact and OVX Female pOka treatment groups. In panel **c** the bar with an asterisk indicates a significant difference (*p* < 0.05) between the Male pOka and the intact Female POka treatment groups. The number of animals in each treatment group was pre-determined and is shown on the figure in parentheses

Because a majority of females suffering from PHN are post-menopausal and have low levels of plasma estradiol the pain response of ovariectomized females was compared to males. Sprague Dawley rats injected with virus (650 pfu/μl) showed a significant response F(1, 144) = 117.0, *p* < 0.001 in the first week but there was no significant sex difference (Fig. 7a). Two weeks after VZV infection the intact and OVX females showed significantly greater behavioral response than males (Fig. 7b). Long Evans rats also had a significant response to VZV infection (360 pfu/μl) in the first week F(1, 144) = 74.5.0, *p* < 0.001 and two weeks after VZV inoculation the intact female and OVX female rats had a significantly greater response than males (Fig. 7d).

### The increased pain response observed in female rats was not the result of greater viral infection

Two weeks of PEAP testing the animals were sacrificed and the ganglia isolated for immunostaining with the VZV marker IE62. The percentage of trigeminal ganglia neurons (NeuN positive) staining for the VZV marker IE62 was 16% ± 1.5 for the intact Sprague Dawley females, 10% ± 1.3 for the OVX females and 28% ± 3.7 for the males. The males had a significantly greater percentage of neurons staining for IE62 (*p* > 0.05) as compared to the females and there was no significant difference between the intact and OVX female groups.

### Aversive behavioral response in cycling females after VZV injection

**Fig. 8:**
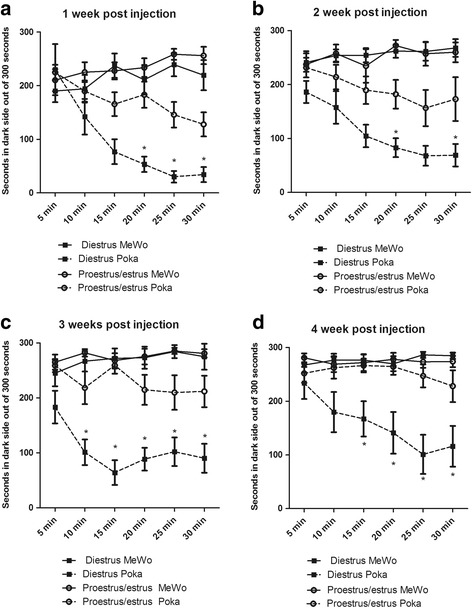
VZV induced and aversive response that was affected by the estrous cycle. Weekly PEAP testing was completed on cycling female rats after VZV injection. A total of 24 rats whose whisker pad was injected with MeWo cells and a total of 25 rats whose whisker pad was injected with MeWo cells infected with 650 pfu/ μl VZV were included in this experiment. Smears were completed on each day of testing and the number of animals at each cycle phase (i.e., diestrus or proestrus/estrus) was a minimum of 8 rats but that number would vary from week to week depending on the estrous cycle. Testing was performed once a week for four weeks after whisker pad injection, in panel **a** testing was completed 1 week after injection, in panel **b** 2 weeks after injection, in panel **c** 3 weeks after injection and in panel **d** 4 weeks after injection. An asterisk indicates a significant difference (*p* < 0.05) between the Diestrus pOka group and the Proestrus/estrus pOka group

The behavioral response after VZV injection of the whisker pad was determined during the estrous cycle. In Fig. 8 the estrus and proestrus data were combined and the diestrus I and diestrus II (i.e., metestrus) data were combined because of their similar estradiol levels (data not shown). A significantly greater response was observed in rats with low estradiol (Diestrus pOka) versus the rats with higher plasma estradiol (Proestrus/estrus pOka) in week one F(1, 281) = 5.8, *p* < 0.05 (Fig. 8a, n≥8), two F(1, 281)= 4.4, *p*<0.05 (Fig. 8b), three F(1, 281)= 61.6, *p*<0.001 (Fig. 8c) and four (1, 281)= 12.4, *p*<0.01 (Fig. 8d). No significant cycle response was observed in weeks five and six (data not shown). During the first F(1, 281)= 19.2, *p*<0.001, second F(1, 281)= 11.2, *p*<0.01, third F(1, 281)= 21.2, *p*<0.001, fourth F(1, 281)= 4.3, *p*<0.05 week there was a significant virus effect. A significant interaction between cycle and virus treatment was observed in week two F(1, 281)= 9.4, *p*<0.01, three F(1, 281)= 21.3, *p*<0.001 and four (1, 281)= 14.6, *p*<0.001. Post-hoc testing indicated a significant decrease in the Diestrus pOka group versus the Diestrus MeWo group at the 15, 20, 25 and 30 min time points in weeks one, two, three, and four. No significant difference was observed in week five and six. These results indicate that the development of pain responses to VZV is influenced by the stage of the estrous cycle.

## Discussion

Mechanical allodynia is the most common, most debilitating and the most difficult to treat component seen in PHN patents. Persistent mechanical allodynia and thermal hyperalgesia are present after VZV inoculation of the footpad [14–20]. The study here expands the footpad model to the orofacial region by demonstrating that VZV induces a significant behavioral response after stimulating the facial region [20, 21, 44]. These responses included thermal and mechanical hypersensitivity, as well as, an aversive response. Interestingly the aversive response, as measured by the PEAP assay [31], lasted several weeks longer than the hypersensitivity measured by Ugo Basile chambers. The longer PEAP response suggests that either the PEAP assay was more sensitive than the Ugo Basile chambers in measuring responses or that the affective component is present longer than the sensory component. In contrast to our results, previous studies injecting compete Freund’s adjuvant into the paw showed that sensory responses (von Frey) and affective responses (PEAP test) lasted for similar times [31]. One explanation for this inconsistency would be that adjuvant induces pain through a different mechanism than VZV. Behavioral tests, such as the PEAP test, require supraspinal pain processing and would be useful in the drug development for patients [36, 37]. In one example of preclinical drug testing, gabapentin reduced the affective PEAP response in this rat model consistent with gabapentin being efficacious in treating patients with HZ associated pain [45]. It is interesting to note that anxiety-like behaviors were not reversed by gabapentin treatment in previous studies [20], but gabapentin does effect the PEAP measurement (reverse the aversive behavior), consistent with the idea that anxiety does not bias the PEAP measurement [36, 46].

Pain is reported 3.75 times more often in women than in men after HZ infection [25] but no model currently exists to study this sex difference. We show here that VZV induced pain was influenced by sex. VZV inoculated female rats experienced the same level of aversive behavior as the VZV injected males post-injection week 1, but by week 2 females clearly experienced a higher level of pain behavior, consistent with females experiencing more orofacial pain-related sensations [47–49]. One reason for the sex difference could be related to sex steroids. Of note is the fact that the males had a greater percentage of IE62 positive neurons but showed less aversive behavior suggesting that viral infection rates do not explain the sex difference. Moreover, our data support a role for sex steroids because during the estrous cycle there was a significantly greater PEAP response in diestrus rats (when estradiol is low) in comparison to proestrus/estrus rats (when estradiol is higher). This data is consistent with the nociceptive response in an inflammatory TMJ model where rats given a diestrus level of estradiol (low) had a greater nociceptive response in comparison to rats given a proestrus dose of estradiol (high) [50]. Mechanistically estradiol has been shown to alter the pain response through G-protein signaling [51]. Moreover, estradiol utilizes G-protein signaling to alter the nociceptive response by changing the responsiveness of N-methyl-D-aspartic acid (NMDA) receptors and by enhancing dendritic spine growth [52]. Although the mechanism is unclear as to why the nociceptive response was higher in females this animal model can be used to explore the mechanisms contributing to the observed sex difference.

Humans with HZ associated pain do not require continued replication of VZV because administration of the antiviral acyclovir (blocks viral replication) does not reduce the pain response [53]. Similarly, VZV induced pain was not ameliorated by antiviral agents in the rat footpad model [54]. As in humans, VZV induced hypersensitivity in rodents can be present without detectable viral replication [44, 55, 56]. Recent work has indicated that VZV goes through a partial replication cycle in animals resulting in translation of viral proteins that can induce pain [53]. Together the data suggests continued production of lytic virus and necrosis of neurons is not a mechanism for induction or maintenance of the pain response [57]. To date, no animal has supported VZV ganglionic reactivation [58], so PHN after reactivation cannot be studied. Interestingly, Dr. Kinchington’s group has recently reported the first reliable experimental VZV reactivation in vitro using human neuron cultures [59] and proposed that both human PHN and pain in rats are the result of VZV gene expression without viral replication. Our studies add to this previous work by extending the pain response to the orofacial region which contributes to pain in up to 28% of herpes zoster patients [22, 23]. Using this novel model of VZV these pain mechanisms can be determined in the orofacial region.

Zoster-associated pain may last 30–90 days during the acute phase however, about 20–30% of individuals develop chronic pain (>90 days) termed post-herpetic neuralgia (PHN), which may last months to years [6–8, 60, 61]. In males a VZV dose of 65,000 pfu/whisker pad resulted in a significant response for one week and a dose of 190,000 pfu/whisker pad resulted in a 7 week response. Assuming a linear relationship between dosage and response an injection of 300,000 pfu/whisker pad would result in a 12 week response. A 12 week or 90 day pain response is one of the diagnostic criteria for PHN [60]. Future studies investigating higher dosages could indicate that this model is applicable for studying the mechanisms resulting in the development of PHN.

VZV infection of the whisker pad resulted in IE62 staining within the V1/V2 and V3 regions of the trigeminal ganglia. Because the V1/V2 branch innervates the whisker pad it was not clear why some IE62 staining was present within the V3 region. This is the first evidence for VZV infection of the trigeminal ganglia after injection into the rodent whisker pad but VZV is a human herpes virus, one of eight types of herpes viruses. Whisker pad injection of herpes simplex virus type 1 (one of the eight types of herpes viruses) resulted in a small amount of infection of the V3 region with a majority of the infection being present in the V1/V2 region [62], consistent with our results. Studies of the somatotopic organization of the trigeminal ganglia indicated that there was overlap in the ganglionic regions that contribute to the V1/V2 and V3 branches [63]. The results could be explained by this overlap; if some of the IE62 positive neurons in the V3 region actually project to the whisker pad then it would be expected that these neurons would become infected by VZV.

## Conclusions

Our study uses behavioral testing to assess zoster associated orofacial pain in the rat model. For the first time a sex difference is shown after VZV injection, consistent with epidemiological evidence that women report PHN more than men [24, 25]. This model can be utilized to study one, the mechanisms of zoster-associated orofacial pain, two, test drug therapies to treat humans effectively and three, study the mechanisms contributing to the sex differences underlying zoster associated pain.

## Abbreviations

VZV: Varicella zoster virus
HZ: Herpes zoster
PHN: Post-herpetic neuralgia
pOka: VZV Parent of Oka
MEM: Minimal Essential Media
FBS: Fetal bovine serum
PEAP: Place Escape/Avoidance Paradigm
Pfu: Plaque forming units
